# Immersive Virtual Reality for Pain and Anxiety Management Associated with Medical Procedures in Children and Adolescents: A Systematic Review

**DOI:** 10.3390/children11080975

**Published:** 2024-08-13

**Authors:** Eloy Sánchez-Caballero, Lucía Ortega-Donaire, Sebastián Sanz-Martos

**Affiliations:** 1 Andalusian Health Service, 23007 Andalusian, Spain; esc00023@red.ujaen.es; 2 Department of Nursing, Faculty of Health Sciences, University of Jaén, 23071 Jaén, Spain; lortega@ujaen.es

**Keywords:** virtual reality, anxiety, pain, child

## Abstract

(1) Objectives: The purpose of this study was to investigate the studies that evaluate the effectiveness of immersive virtual reality (VR) as a distraction technique for pain and anxiety associated with medical procedures in children and adolescents. (2) The methods involved a systematic review of randomized controlled trials retrieved from databases in health sciences (Pubmed, CINHAL, Scopus, WOS, ProQuest, Cuiden Plus, InDICEs-CSIC). PRISMA guidelines were followed. (3) Results: Twelve trials were included. Four involved venipuncture, four involved surgical procedures, one involved vaccination, one involved burn care, one involved secondary wound closure, and the last one involved subcutaneous port access. (4) Discussion: Children who undergo medical procedures often experience pain and anxiety, which affects their physical condition and their relationships with caregivers and health professionals. Immersive VR is an effective alternative to medications to help in these cases. No author found statistically significant differences against the use of VR for distraction and palliation of pain and anxiety, which seems to be more effective at a younger age. It is important to personalize the immersive VR experience and equipment. (5) Conclusions: VR, when used with analgesics and anesthetics, appears effective in managing pain and distress caused by medical procedures.

## 1. Introduction

In 2020, the International Association for the Study of Pain defined pain as “an unpleasant sensory and emotional experience associated with, or resembling that associated with, actual or potential tissue damage”, clarifying that it normally plays an adaptive role and is not the same as nociception, but is a personal concept defined by the individual’s own experience, who can not only manifest it verbally but also through non-verbal language [1]. Pain often causes anxiety, fear, and/or stress, so both the intensity of pain and anguish must be taken into account in its management [2]. Likewise, in 2018, the Virtual Health Library defined pain associated with medical procedures as “pain associated with examination, treatment or procedure” [3].

During childhood and adolescence, children undergo medical procedures that may generate pain and distress. For example, from birth until reaching the age of eighteen, the Spanish Association of Pediatrics recommends the immunization of children and adolescents with up to twenty-eight doses of transcutaneous vaccines [4]. It is estimated that 32% of infants (<1 year of age), 17% of children (1–10 years of age), and 38% of adolescents (11–18 years of age) who are hospitalized patients experience severe to moderate pain, altering their physical and mental well-being [5]. This is true before, during, and after the painful procedure, influencing future pain thresholds and coping mechanisms in adulthood [2]. Ineffective pain management affects the performance and satisfaction of primary caregivers and healthcare personnel [2].

On the other hand, according to the Diagnostic and Statistical Manual of Mental Disorders, in its fourth version (DSM-V), anxiety is “the psychological condition associated with intense worry and/or fear in response to a specific stimulus and the absence of an adaptive reaction to it” [6]. In a surgical setting, children can feel intense anxiety, which affects their mental and physical health, favoring the appearance of postoperative adverse effects, delaying recovery in the postoperative period, hindering the child’s self-care and/or even increasing the intensity of pain in the immediate postoperative period, which in turn can imply tripling the consumption of analgesics. There is a tendency to medicate children prior to surgery to alleviate future pain and anxiety; however, this practice can trigger nausea and vomiting if analgesics were administered, or postoperative delirium, agitation, and even more pain if anesthetics were administered. The use of interventions based on play and audiovisual distraction is a novel concept that could be effective in the preoperative period, although there is still controversy [7].

In 2011, Stevens et al. [8] observed that 78.2% of their sample of 3822 hospitalized children and adolescents had undergone at least one painful medical procedure the day before data collection, so on the same day they collected their data, 78.1% of these subjects received some type of analgesic intervention: mixed (32.3%), psychological (25%), physical (26.1%), and pharmacological (84.8%) interventions. The most commonly used drugs were paracetamol (70.6%), opioid narcotics, NSAIDs [5], adjuvants (ketamine and clonidine), local anesthetics, and sucrose. The disadvantages of a pharmacological approach include adverse effects, reluctance of medical professionals to prescribe opioid analgesics, their high cost, and drug shortages in developing countries [7,8,9].

The evidence supporting the use of non-pharmacological measures complementary to pharmacological ones for pain management is abundant. They can be classified into physical (repositioning, cryotherapy, thermotherapy, contrast baths, non-nutritive suction) and psychological (education, pre-procedure preparation, storytelling, music, video games, hypnosis, television viewing, cognitive-behavioral therapy, breathing–relaxation, and distraction exercises). In the active techniques, the child must participate. Some examples include interactive toys, video games, and virtual reality (VR). In passive techniques, the child’s attention is captured passively, without involving them in any interactive activity, such as listening to music or watching TV [10].

As a result, interactive distraction strategies are gaining ground over passive ones. VR is one of the most recent techniques, and it is gaining momentum due to its origin in new technologies. If proven effective, it could constitute a non-pharmacological alternative for its application in pediatric users [2].

We can define virtual reality (VR) as a platform that provides an artificial environment where the user can perceive the simulation created by the system and see it as real. Virtual reality can be immersive, semi-immersive, or non-immersive. Immersion is based on a three-dimensional environment where the user’s physical reality is replaced by an artificial environment. On the other hand, non-immersive devices show the environment on the screen. In both cases, it is possible to interact with the virtual environment through input peripherals or through body movements. Semi-immersive virtual reality is the exact middle ground between the previous levels of immersion, generally allowing users to experience in a three-dimensional virtual environment while remaining connected to real-world sights, sounds, smells, and physical objects [11,12]. VR was developed in the second half of the 1990s for military maneuvers, although it soon moved to the civilian world for recreational and therapeutic purposes [13]. The multisensory inputs (tactile, auditory, and visual) of VR provide the subject with an immersive experience in a fictitious virtual environment, in which he or she can participate. Thus, the immersive VR wearer does not perceive the real world. This is an advantage of VR over other non-pharmacological methods of pain relief. However, this is not the only objective of treatment for which VR has been intended; for example, VR exposure therapy is used for phobias and social disorders [14].

Immersive VR interventions vary according to the equipment used, the virtual world, and the level of participation required from the user. The complete equipment consists of VR goggles, headsets, hand-held control devices, hardware (cell phone), and computer software [2,15].

There is still uncertainty about the efficacy of VR as a non-pharmacological pain distraction measure, and its application in clinical practice has been questioned due to its cost, the relatively bulky equipment involved, the technological expertise it again requires, and the risk of cyber-disease, which is a discomfort that can occur after a few minutes of using a virtual reality device, as a result of conflicting sensory information received by the brain. Symptoms include dizziness, nausea, eye fatigue, sweating, and headaches [2,16]. While some authors have published Cochrane reviews on the management of acute pain with VR in children [2], others have focused on the control of distress and pain associated with medical procedures requiring needles using psychological interventions in children and adolescents [17]. Therefore, this systematic review aims to examine the literature on the use of immersive VR for managing pain and anxiety associated with medical procedures in both children and adolescents. In addition, it seeks to describe the advantages and disadvantages of the use of immersive VR in the clinical care settings for managing pain and anxiety and to identify alternatives to immersive VR when this is not an option.

## 2. Materials and Methods

The literature search for this systematic review was conducted between November 2022 and January 2024. Descriptors were extracted from the DeCS/MeSH thesaurus of the Virtual Health Library: “Treatment Outcome”, “Virtual Reality”, “Anxiety”, “Pain”, “Procedural”, “Child”, and “Adolescent”. The individual search strings for each database are listed in Table 1.

Randomized clinical trials (RCTs) whose experimental group intervention and object of study were VR were included. No language restrictions were applied. Articles were selected in which any of the study’s keywords appeared in the title and/or abstract. We excluded those studies in which the pain and anxiety of the sample were not associated with medical procedures, and those in which the ages of the study subjects were not between 6 and 18 years.

We searched PubMed, CINAHL Complete, Scopus, ProQuest, Web of Science, Cuiden Plus, and InDICEs-CSIC. Institutional access to the University of Jaen Library was used. On one occasion, the full-text article was requested from the author.

The selection of the studies that could be included in the review was carried out in parallel by the three authors of the manuscript, seeking to find an index of agreement between them of more than 0.8 points, evaluated by the Kappa index. Studies with less than the critical value or with values in the confidence interval of 0.7 points or lower were eliminated from the selection. The Kappa index was calculated with JASP 0.17 version for Windows.

The Critical Appraisal Skills Program (CASPe) and the PEDro scale were used to assess the methodological quality of the results [18]. In order not to be excluded, the RCTs had to meet the first three items of the CASPe guide with an affirmative response (Table 2 and Table 3). As a result of the evaluation, three articles were eliminated.

**Figure 1:**
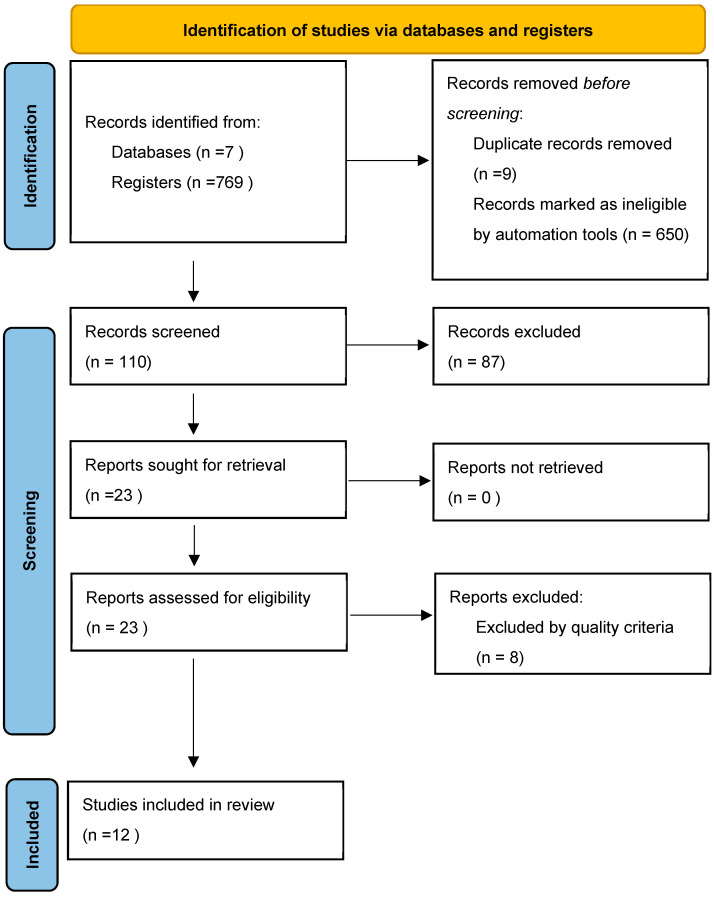
Flowchart of the study selection process, adapted from the PRISMA model.

This review followed the criteria for reporting systematic literature reviews and meta-analyses, as defined by the Preferred Reporting Items for Systematic Review and Meta-Analyses (PRISMA) statement [32]. Abstract screening, full-text review, and data extraction were then conducted in accordance with the PRISMA guidelines. Figure 1 illustrates the PRISMA flowchart used to guide the systematic review process.

## 3. Results

Prior to data extraction, an analysis of the potential biases present in the selected studies was carried out using the Rob2 tool (Supplementary Material S2).

According to the search criteria, 769 results were returned, and after passing the filtering and quality control process, 12 articles were finally included. Table 4 shows the characteristics of the studies included in this review, classified according to the painful and/or anxiogenic medical procedure.

With regard to the characteristics of the RV of the trials, they were variable. The list of equipment, software, and cost of the RV is shown in Table 5, which was drawn up by the authors.

The frequency with which the questionnaires and scales chosen by the authors were used to measure the variables of pain intensity and level of anxiety, self-perceived by the sample, caregivers, parents and/or health professionals, or observed in the sample by caregivers, parents and/or health professionals, is shown in Table 6. The blue-shaded boxes indicate responses provided by the study subjects, while the green-shaded boxes indicate responses based on the observations of the evaluators.

Of the twelve included trials, eleven measured both variables, and one, by Jung et al. [25], only estimated the level of anxiety observed in the study population.

On the other hand, eight articles found statistical significance in favor of RV as a measure of interactive distraction from pain and anxiety associated with the medical procedure [12,15,19,21,25,26,27,30]. Gershon et al. [12] documented the statistical significance between the VR and passive distraction groups versus the control group (*p* ˂ 0.05). Jeffs et al. [27] only found a significant difference between the passive distraction group vs. the VR group, but not vs. the standard of care (SOC) group (APPT-WGRS8; +9.7 mm; 95% CI: [−9.5, 28.9]; *p* = 0.32).

Four studies yielded null results (*p* > 0.05), finding no statistical significance in the differences between the RV group versus the control group [20,23,24,31]. None of the included articles showed unfavorable results for the use of VR on the intensity of acute pain and associated anxiety.

In addition, four RCTs of the twelve included assessed anxiety of the subjects’ primary caregivers as a secondary outcome. Two found statistically significant differences between the caregivers in the VR group and the control group: Chang et al. [30] (difference before and after vaccination VAS9; VR vs. control: −4 points vs. 0 points; *p* = 0.009) and Liu et al., [26] (during the SUDS procedure10; VR vs. control: 11.50 mm ± 17.67 mm vs. 27.39 mm ± 30.48 mm, *p* = 0.041; r = 0.28). While the other two trials, by Jung et al. [25] and Eijlers et al. [24], yielded null results (*p* > 0.05).

Of the twelve included trials, six RCTs measured parents’ and/or healthcare staff’s satisfaction and willingness to return to VR. Two trials found a statistical significance in favor of VR use when comparing the responses of the control group versus the VR group when asked about their satisfaction; Jung et al. [25] estimated the difference in parent VR vs. no VR satisfaction to be −3 points (95% CI [−8, 2]; *p* = 0.15); and Gold et al. [15] found satisfaction in children (*p* ˂ 0.01) and in parents and nursing staff (*p* > 0.05).

The trials by Clerc et al. [23] and Chang et al. [30] recorded the opinions of nursing and medical staff in the intervention group on VR; all professionals considered VR a simple and acceptable tool that they would use again. Liu et al. [26] sought the opinions of children and the caregivers of those who had previously undergone nasal endoscopy. They found that both groups preferred the use of VR to relieve discomfort over standard care and would choose it again for future similar procedures; their caregivers were of the same opinion, except for one, who represented 3.33% of the total caregivers in the VR group, and who was unsure whether they would prefer it again over standard care.

Eight RCTs documented the absence or presence of unwanted side effects with the use of VR [15,19,20,23,26,27,30]. A total of five subjects in the VR groups experienced adverse effects: two reported headaches and nausea and three mild dizziness and nausea; they were resolved by removing the VR helmet. In contrast, seven patients in the SOC group of the Chan et al. trials [19] reported dizziness, nausea, and headache, of whom four vomited.

Table 7 shows the results of the evaluation of the levels of evidence assessed using the GRADE scale.

## 4. Discussion

The results of this review show the effect of VR on the intensity of pain, anxiety, and distress associated with medical procedures, alleviating and reducing them, except for those occasions when the authors found no statistical significance. Of the RCTs that yielded null results, the Goldman et al. trial [31] was the only one of the selected studies that studied the use of VR during wound closure by secondary intention. In this regard, while it is true that the VR intervention proved to be more effective than standardized usual care, it is difficult to establish its added value over other distraction formats because the standard of usual care was often not well defined. Therefore, this study was inconclusive as to the effect size of VR versus other forms of preparation and distraction from pain and anxiety associated with medical procedures.

This effect of VR is accentuated when pain and anxiety variables are reported based on children’s behavior, as observed by caregivers and nursing staff. Furthermore, in as many as six RCTs, younger children were observed to benefit more from VR distraction than older children [12,19,23,26,30]. In their trial, Clerc et al. [23] observed that VR might be more effective between 6 and 8 years of age than between 13 and 16 years of age, although statistical analysis was not performed because the sample was too small. It could be due to the predominant magical thinking of children, the greater immersion in the virtual world of the software, and the higher degree of anticipatory anxiety and distress experienced at younger ages. In contrast to this finding, Schlechter et al. [20] observed that among children around 7 years of age there were more children who did not tolerate VR compared to those around 13 years of age.

Another factor that modulates the impact of virtual reality on the pain experience is immersion in the virtual world. The more a child interacts with video games, the less intense the perception of pain becomes because the child becomes more distracted and disconnected from the real world [32,33]. Thus, it is essential to distinguish between whether the software allows the user to reposition and reorient himself on the map, as well as choose the point of view and field of vision when this is not the case, as is the case with videos previously programmed to be viewed with VR glasses, limiting immersion in the virtual environment and forcing the user to follow the progress of the content creator. The role of immersion should be the subject of study in future research [34,35].

Along these lines, all the VR equipment the test authors chose for their research included audiovisual and tactile sensory inputs; that is, modalities that allowed children to interact in audiovisual and tactile ways with what they were viewing through the VR equipment. Clerc et al. [23] programmed the Roller Coaster application because of the large amount of visual stimuli it presents to the user to capture their attention, the control of the field of view, the ease of restarting the game with another user, the possibility of playing it in the supine position and because it is an offline application with no ads. The interactive game presented by Jung et al. [25] to their VR group was specifically designed for perioperative use in the pediatric population. Liu et al. [26] downloaded a video game that, far from promoting physical movement in children, was designed to keep children’s heads still during endoscopy. Gershon et al. [12] presented the virtual environment to both study groups; however, the standard care group was only shown on the monitor and the VR group on the VR device; the differences they found reinforce the intention to include as many and as high-quality sensory inputs as possible to attract the child’s attention.

Alternatives to invasive procedures are costly, whether in terms of expenses, time, or biological side effects [33,36]. In this review, alternatives to VR have been identified for those cases in which it is not indicated. They should be pleasurable activities that have been previously negotiated with the child. Creating a music playlist, painting, playing board games and video games, watching movies, interacting with humanoid robots, and/or opening mobile applications. Mobile applications to the child’s liking and, if possible, interactive applications are suggested to help them concentrate and complement traditional anxiolytic and analgesic methods. Non-pharmacological measures encourage social interaction and communication skills, protect from fear, and strengthen the bond of the healthcare professional with the patient during the painful and anxiogenic procedure [6,34,37].

The following limitations should be taken into account when interpreting the results. First, the retrieved RCTs constitute a heterogeneous sample of the literature because of the age of the subjects, the methodological quality, the assessment of biases, the VR software, and the medical procedure on which the VR was intended to be tested. For this last reason, one should cautiously expect varying degrees of effectiveness of VR on pain intensity, depending on the specific procedure the child is to undergo; even so, reading the results as a whole provides a global view that should be considered, since for some painful procedures there is insufficient evidence to support the use of VR [31]. A more in-depth analysis of the results, by means of a meta-analysis, was not possible due to the wide variability in outcome variables and the scarcity of studies for each one, with high variability present. As a future line of research, we suggest expanding the number of studies that allow a more in-depth evaluation.

In addition to this, ignoring the subjectivity of the experience of pain and anxiety, and assuming the proper practice of the health professional who followed the instructions to measure the variables using different scales and validated questionnaires, both self-administered and hetero-administered, and with the participation of various people (parents, caregivers, nursing staff, physicians, and researchers) who completed the questionnaires and scales, a great difficulty arose while performing the statistical analysis of the results in this systematic review [12,15,19,20,21,24,25,26]. This makes it difficult to draw clear conclusions.

On the other hand, the administration of medications and anesthetic or analgesic products prior to the procedure decreased the subjects’ pain intensity and could have biased the results. In both groups, the children might have felt less pain than they would have felt without their pharmacologic management, and the difference in the results of the study variables between the VR and standardized care groups might be greater [20,23,31]. However, in all trials, the samples were not inhomogeneous for this reason. Of the nine trials that administered anesthesia and analgesia to their sample, those by Schlechter et al. [20], Clerc et al. [23], and Goldman et al. [31] found no statistically significant difference between the outcomes of the standardized care groups versus the VR groups. The remaining authors found significant differences in favor of the use of VR as a distractor measure of pain and anxiety associated with medical procedures. These data support the complexity of pain perception and the importance of addressing the emotional component, even when attempting to interfere with nociception. Without addressing anticipatory anxiety and distress to help children and adolescents cope with their stressful situations, they will continue to experience pain [15].

On the other hand, although all the selected RCTs effectively included a description of the VR equipment used, only three of them performed a cost–benefit analysis [20,23,31], and none of them studied the feasibility of its implementation in the local healthcare system. For this reason, it was not possible during this review to deduce the feasibility of introducing VR in the health systems of our environment.

On the one hand, although VR alters the perception of pain caused by medical procedures and the associated anxiety, especially in younger children, it is more effective if introduced prior to the painful procedure to reduce anticipatory anxiety, which increases pain and distress [19,30,31]. It is an easy-to-use and tolerable tool for the pediatric population.

Authors Chan et al. [19] recorded comments from participants and healthcare professionals in the study about the use of VR that may be of great value if trying to implement this tool in clinical practice: “I have PlayStation VR at home, this was not that exciting”, “it increases the pain if the patient cannot see the cause”, “it made the experience easier. Last time we had to hold it”, ‘he wouldn’t freak out if he didn’t see the needle’, ‘for a mother whose son has regular blood tests and autism, this was awesome’, ‘he always wants to see the needle going in to be calmer’, ‘it usually takes three people to hold him’, ‘please make sure the mask is comfortable’, “I would recommend giving time to process the needle procedure and wear the headset prior to venipuncture. Especially in children with autism”, ‘the helmet was dropped a bit’, ‘better if the child had been given a game they were familiar with’, ‘would be more inclusive in other languages’, ‘I spent time preparing the children about the goggles’, “seemed surprised when the needle was inserted. I would have warned earlier perhaps”, “the patient commented that he was bored with the video. Provide more age appropriate content”.

## 5. Conclusions

In conclusion, this systematic review shows that children and adolescents undergoing painful or anxiogenic health care procedures benefit from VR as a non-pharmacological distraction method, complementary to traditional pharmacological methods, for both pain and associated anxiety. No conclusion has been reached regarding secondary outcomes, such as the number of venipuncture attempts, postoperative delirium, rescue analgesia, or the cost–benefit ratio. Even so, more research is needed in this area.

## Figures and Tables

**Table 1:** Databases, search strings and selected articles.

Databases	Search Strategy	No. of Documents Returned (n=)	Discarded Items (n=)	Final Sample of Documents (n=)
PUBMED	(Treatment Outcome[mh] OR Treatment Outcome[tiab] OR Treatment Effectiveness[tiab] OR Treatment Efficacy[tiab] OR Clinical Effectiveness[tiab] OR Clinical Efficacy[tiab]) AND (Virtual Reality[mh] OR Virtual Reality[tiab] OR Reality Virtual[tiab]) AND (Anxiety[mh] OR Anxiety[tiab] OR Nervousness[tiab] OR “Pain, Procedural”[mh] OR Pain Procedural[tiab] OR Procedural Pain[tiab]) AND (Child[mh] OR Child[tiab] OR Children[tiab] OR Adolescent[mh] OR Adolescent[tiab] OR Adolescents[tiab] OR Adolescence[tiab] OR Teen*[tiab] OR Teenager*[tiab] OR Youth*[tiab])	No filters: 36 With filters: 23	Title: 13 Abstract: 1 Full text: 1	8
CINAHL Complete	(MH “Treatment Outcomes” OR AB “Treatment Effectiveness” OR AB “Treatment Efficacy” OR AB “Clinical Effectiveness” OR AB “Clinical Efficacy”) AND (MH “Virtual Reality” OR AB “Virtual Reality” OR AB “Reality Virtual”) AND (MH “Anxiety” OR AB “Nervousness” OR MH “Pain, Procedural” OR AB “Procedural Pain”) AND (MH “Child” OR AB “Children” OR MH “Adolescence” AB “Adolescent*” OR AB “Teen*” OR AB “Teenager*” OR AB “Youth*”)	No filters: 15 With filters: 5	Title: 2 Abstract: 0 Full text: 0	3
SCOPUS	(“Treatment Outcome” OR “Treatment Effectiveness” OR “Treatment Efficacy” OR “Clinical Effectiveness” OR “Clinical Efficacy”) AND (“Virtual Reality” OR “Reality Virtual”) AND (Anxiety OR Nervousness OR “Pain, Procedural” OR “Procedural Pain”) AND (Child OR Children OR Adolescent OR Adolescents OR Adolescence OR Teen* OR Teenager* OR Youth*)	No filters: 80 With filters: 63	Title: 49 Abstract: 1 Full text: 1	12
WOS	TS = (“treatment outcome*”) AND TS = (“Virtual Reality”) AND TS = (Anxiety OR “Pain, Procedural”) AND TS = (Child* OR Adolescent)	No filters: 65 With filters: 0	Title: 0 Abstract: 0 Full text: 0	0
PROQUEST	(MESH “Treatment Outcome” OR AB “Treatment Effectiveness” OR AB “Treatment Efficacy” OR AB “Clinical Effectiveness” OR AB “Clinical Efficacy”) AND (MESH “Virtual Reality” OR AB “Reality Virtual”) AND (MESH Anxiety OR AB Nervousness OR MESH “Pain, Procedural” OR AB “Procedural Pain”) AND (MESH Child OR AB Children OR MESH Adolescent OR AB Adolescents OR AB Adolescence OR AB Teen* OR AB Teenager* OR AB Youth*)	No filters:562 With filters: 8	Title: 8 Abstract: 0 Full text: 0	0
CUIDEN PLUS	(“Realidad Virtual”) AND (“Dolor Asociado a Procedimientos Médicos” OR “Ansiedad”) AND (“Niño” OR “Adolescente”)	No filters: 1 With filters: 1	Title: 0 Abstract: 0 Full text: 1	0
ÍnDICEs-CSIC	“realidad virtual” AND “dolor”	No filters: 10 With filters: 10	Title: 6 Abstract: 1 Full text: 3	0

Source: author’s own elaboration.

**Table 2:** Evaluation of the quality of search results using the CASPe guide.

*Investigation*	*1*	*2*	*3*	*4*	*5*	*6*	*7*	*8 IC%*	*9*	*10*	*11*
Gershon, J. et al., 2004 [12]	Yes	Yes	Yes	No	NM	Yes	η^2^ = 0.09	NM	No	Yes	Yes
Gold, J.I. et al., 2006 [15]	Yes	Yes	Yes	No	Yes	Yes	*r_xy_* = 0.68–0.96	NM	No	Yes	Yes
Chan, E. et al., 2019 [19]	Yes	Yes	Yes	No	Yes	Yes	NM	95	No	Yes	Yes
Schlechter, A.K. et al., 2021 [20]	Yes	Yes	Yes	No	Yes	Yes	NM	NM	No	Yes	Yes
Özalp, G. et al., 2020 [21]	Yes	Yes	Yes	No	Yes	Yes	*R*^2^ = 0.041–0.341	NM	No	Yes	Yes
Lee, H.N. et al., 2021 [22]	Yes	No	-	-	-	-	-	-	-	-	-
Clerc, P.G.B. et al., 2021 [23]	Yes	Yes	Yes	No	Yes	Yes	*R*^2^*_N_* = 0.077; 0.092	95	No	Yes	Yes
Eijlers, R. et al., 2019 [24]	Yes	Yes	Yes	No	Yes	Yes	NM	NM	No	Yes	Yes
Jung, M.J. et al., 2021 [25]	Yes	Yes	Yes	No	Yes	Yes	NM	95; 97.5	No	Yes	Yes
Liu, K.Y. et al., 2021 [26]	Yes	Yes	Yes	No	Yes	Yes	*r_xy_* = 0.28–0.75	NM	No	Yes	Yes
Jeffs, D. et al., 2014 [27]	Yes	Yes	Yes	No	Yes	Yes	NM	95	No	Yes	Yes
Das, D.A. et al., 2005 [28]	Yes	No	-	-	-	-	-	-	-	-	-
Russo, L. et al., 2022 [29]	Yes	No	-	-	-	-	-	-	-	-	-
Chang, Z.Y. et al., 2022 [30]	Yes	Yes	Yes	No	Yes	Yes	NM	95	No	Yes	Yes
Goldman, R.D. et al., 2021 [31]	Yes	Yes	Yes	No	Yes	Yes	NM	NM	No	Yes	Yes

NM = not shown. 1: Is the trial oriented to a clearly defined question? 2: Was the allocation of patients to treatments randomized? 3: Were all patients who entered the study adequately considered until the end of the study? 4: Was blinding maintained? 5: Were the groups similar at the start of the trial? 6: Were the groups treated similarly at the start of the trial? 6: Were the groups treated equally regardless of the intervention under study? 7: Is the treatment effect very large? 8: What is the precision of this effect? 9: Can these results be applied in your environment or local population? 10: Were all clinically important outcomes taken into account? 11: Do the benefits to be obtained justify the risks and costs? Source: author’s own elaboration.

**Table 3:** Evaluation of the quality of search results using the PEDro scale.

Article	1	2	3	4	5	6	7	8	9	10	11
Gershon, J. et al., 2004 [12]	Yes p.2	Yes p.2	Yes p.2	Yes p.3	No p.6	No p.6	No p.6	Yes p.4	Yes p.4	Yes p.4	Yes p.4
Gold, J.I. et al., 2006 [15]	Yes p.2	Yes p.2	Yes p.2	Yes p.3	No	No	No	Yes p.4	Yes p.4	Yes p.4	Yes p.4
Chan, E. et al., 2019 [19]	Yes p.2	Yes p.2	Yes p.2	Yes p.3	No p.2	No p.2	No p.2	Yes p.4	Yes p.4	Yes p.4	Yes p.4
Schlechter, A.K. et al., 2021 [20]	Yes p.2	Yes p.2	Yes p.2	Yes p.3	No p.4	No p.4	No p.4	Yes p.3	Yes p.3	Yes p.3	No
Özalp, G. et al., 2020 [21]	Yes p.6	Yes p.6	Yes p.6	Yes p.10	No p.8	No p.8	No p.8	Yes p.11	Yes p.11	Yes p.11	Yes p.11
Clerc, P.G.B. et al., 2021 [23]	Yes p.2	Yes p.2	Yes p.2	Yes p.4	No	No	No	Yes p.4	Yes p.4	Yes p.4	Yes p.4
Eijlers, R. et al., 2019 [24]	Yes p.2	Yes p.2	Yes p.2	Yes p.5	No	No	No	Yes p.6	Yes p.6	Yes p.6	No
Jung, M.J. et al., 2021 [25]	Yes p.3	Yes p.3	Yes p.3	Yes p.6	No p.8	No p.8	No p.8	Yes p.6	Yes p.6	Yes p.6	Yes p.6
Liu, K.Y. et al., 2020 [26]	Yes p.2	Yes p.3	Yes p.3	Yes p.8	No p.7	No p.7	No p.7	Yes p.3	Yes p.3	Yes p.3	Yes p.3
Jeffs, D. et al., 2014 [27]	Yes p.3	Yes p.3	Yes p.3	Yes p.6	No p.10	No p.10	No p.10	Yes p.6	Yes p.6	Yes p.6	Yes p.7
Chang, Z.Y. et al., 2022 [30]	Yes p.3	Yes p.4	Yes p.4	Yes p.7	No p.9	No p.9	No p.9	Yes p.7	Yes p.7	Yes p.7	Yes p.8
Goldman, R.D. et al., 2021 [31]	Yes p.1	Yes p.2	Yes p.2	Yes p.3	No	No	No	Yes p.2	Yes p.2	Yes p.2	No

**Table 4:** Characteristics of the studies included in the review.

Intervention	Authors and Year	Design	n	Main Results
**Venipuncture**	Chan et al., 2019 [19]	RCT	123	The VR group reported lower levels of pain (−1.78 †; 95% CI, [−3.24, −0.32]; *p* = 0.018) and anxiety (−1.75 †; 95% CI: [−3.09, −0.40]; *p* = 0.01) after venipuncture compared to the SOC group. Caregivers of subjects assigned to the VR group scored children’s distress lower compared to those in the SOC group (VR vs. SOC: 1.0 vs. 4.0; *p* = 0.02).
	Schlechter et al., 2021 [20]	RCT	115	There were no significant differences (*p* > 0.05) in the number of venipuncture attempts, time of venipuncture, changes in pain, and anxiety of children and their parents between the time before and after the procedure between the control and RV groups. The mean age of those children who did not tolerate VR was 7.4 [6.2, 11.1] vs. 12.6 [9.3, 15.6] of those who did (*p* = 0.02).
	Özalp et al., 2020 [21]	RCT	136	Children in the VR-roller coaster and VR-ocean reef groups reported and showed less pain (*p* = 0.00), fear, and anxiety (*p* = 0.00) before and after blood collection compared to the control group. No differences were found between both VR groups (*p* > 0.05).
	Gold et al., 2006 [15]	RCT	20	The control group reported a statistically significant increase in pain due to venipuncture (t = −1.00; *p* > 0.05), while the VR group did not (t = −3.25; *p* < 0.05). A significant relationship was established between the use of VR and pain intensity (r = 0.82; *p* < 0.01).
**Surgical intervention**	Clerc et al., 2021 [23]	RCT	64	No statistical significance was found in the levels of pain (*p* = 0.60) and anxiety (*p* = 0.19) of both groups between pre- and post-QI values. There was a statistical significance in the duration of IQ (VR vs. SOC: 22 min vs. 29 min; *p* = 0.002).
	Eijlers et al., 2019 [24]	RCT	121	No significant differences were found between the VR and SOC groups for the variables of pain, anxiety, and postoperative delirium in the children, as well as anxiety in the parents (*p* > 0.05).
	Jung et al., 2021 [25]	RCT	71	The control group reported greater increases in anxiety upon entering the operating room (5.0 †; 97.5% CI: [2.0, 8.0]; *p* < 0.001) and during induction to general anesthesia (13.3 †; 97.5% CI: [3.7, 23.0]; *p* < 0.001) than the VR group, compared to the measurements taken in the preoperative area.
	Liu et al., 2020 [26]	RCT	53	Subjects in the VR group perceived less pain (*p* = 0.018), anxiety (*p* = 0.0002), and distress (*p* = 0.0001) during endoscopy, and reported greater satisfaction (*p* = 0.0002) than the control group. Caregivers in the RV group reported less anxiety during the procedure (*p* = 0.041).
**Burn care**	Jeffs et al., 2014 [27]	RCT	28	The passive distraction group reported higher levels of pain during wound care than the VR group (+23.7 mm †; 95% CI: [2.4, 45.0]; *p* = 0.029), while the SOC group did not show a significant difference (*p* = 0.32). The VR group was the only group that reported less pain before the procedure than during the procedure.
**Subcutaneous access to central line**	Gershon et al., 2004 [12]	RCT	59	Children distracted with VR showed lower heart rate during access to the implanted device port, and the nursing staff observed fewer signs of pain (*p* < 0.05). The control group showed more signs of distress than the VR group and the no-VR group (*p* < 0.05).
**Vaccination**	Chang et al., 2022 [30]	RCT	30	Children’s reported pain (*p* = 0.04) and fear (*p* = 0.02), as well as their parents’ perceived anxiety (*p* = 0.009) about vaccination were significantly lower in the VR group in the per-protocol analysis. There was no change in nursing staff anxiety between groups (*p* = 0.81).
**Closure by second intention**	Goldman et al., 2021 [31]	RCT	62	No differences were found between the children in the VR group and those in the SOC group for the variables of pain (*p* = 0.458) and anxiety (*p* = 0.890) after suturing their wounds. The control group made more positive and fewer negative comments than the VR group (*p* = 0.10).

CI = confidence interval; SOC = standard of care, usual standard care. †: Comparison between RV group/s vs. control. Source: prepared by the authors.

**Table 5:** Feature of virtual reality.

*Authors and Year*	*VR Features*	Coste
Chan, E. et al., 2019 [19]	Google Pixel XL + Google Daydream. Underwater adventure software.	ND
Schlechter, A.K. et al., 2021 [20]	VR headset + VR glasses + iPhone + headphones. VR Software.	899$
Özalp, G. et al., 2020 [21]	Samsung Galaxy S5 Note + HMD Samsung Gear Oculus mobile phone. Roller Coaster software or Ocean Rift software.	ND
Gold, J.I. et al., 2006 [15]	5DT HMD 800 + control + headset + laptop. Street Luge software.	ND
Clerc, P.G.B. et al., 2021 [23]	HMD VOX+ Z3 3D + mobile phone Asus Zenfone 2 ZE551ML. Roller Coaster software.	* 30$
Eijlers, R. et al., 2019 [24]	HTC Vive HMD + computer. Customized software with virtual operating room environment.	ND
Jung, M.J. et al., 2021 [25]	Samsung Gear VR. Interactive game.	ND
Liu, K.Y. et al., 2020 [26]	Oculus Go VR Glasses + controller + headset. SpaceBurgers™ software.	ND
Jeffs, D. et al., 2014 [27]	VR Glasses Kaiser Optics SR80a + Headset Bose Quiet Comfort 3+ Kensington orbit trackball + PC + tripod; 80° vision. 2003 version SnowWorld software.	ND
Gershon, J. et al., 2004 [12]	VR device + headset + joystick + monitor. Virtual Gorilla software.	ND
Chang, Z.Y. et al., 2022 [30]	HMD Oculus Quest. Viewing angle 100°. SILVER 2 min software.	ND
Goldman, R.D. et al., 2021 [31]	ReTrak Utopia 360 HMD VR + Asus Zenfone 2 ZE551ML mobile phone. VR Roller Coaster application.	220$

ND = not displayed; VR = virtual reality. HMD = head-mounted display; SILVER = soothing immunization leveraging on virtual reality experience. * Cost of the VR headset, not showing the cost of the rest of the components. Source: author’s own elaboration.

**Table 6:** Frequency of use of the instruments for assessing the intensity of pain and anxiety.

*Questionnaire or Scale*	*Chan, E. et al., 2019 [19]*	*Schlechter, A.K. et al., 2021 [20]*	*Özalp, G. et al., 2020 [21]*	*Gold, J.I. et al., 2006 [15]*	*Clerc, P.G.B. et al., 2021 [23]*	*Eijlers, R. et al., 2019 [24]*	*Jung, M.J. et al., 2021 [25]*	*Liu, K.Y. et al., 2020 [26]*	*Jeffs, D. et al., 2014 [27]*	*Gershon, J. et al., 2004 [12]*	*Chang, Z.Y. et al., 2022 [30]*	*Goldman, R.D. et al., 2021 [31]*	Frequency of Use in Variables (f1); Tests (f2)
**FPS-R**	2	1		1	1	1					1	1	*9*; *8*
		1											
**VAT**	2		1										*3*; *3*
**EVA**	2			1		1				2	* 2		*12*; *6*
										4			
**WB faces**			1	1				1					*6*; *3*
			3										
**CASI**				1									*1*; *1*
**SUDS**								1					*2*; *1*
								* 1					
**CEMS**								1					*1*; *1*
**mYPAS**						1	1						*2*; *2*
**STAIC**						* 1	* 1		^†^ 1				*3*; *3*
**VSA**					1							1	*2*; *2*
**APPT-WGRS**									1				*1*; *1*
**CFS**			1								1		*2*; *2*
**Likert Scale**		1											*2*; *1*
		1											
**FLACC**						1							*1*; *1*
**CHEOPS**										1			*1*; *1*

**FPS-R** = Faces Pain Scale-Revised (10 faces, 0–10: 0, no pain and 10 unbearable pain ever felt); **VAT** = Visual Analogue Thermometer, (0–10: 0, no anxiety and 10 anxieties ever felt). **VAS** = Visual Analogue Scale (0–10: 0, no pain/anxiety and 10 unbearable pain/anxiety ever felt); **WB faces** = Wong–Baker Faces Pain Rating Scale (0–10: 0, happy face, no pain and 10, crying face, unbearable pain ever felt); **CASI** = Childhood Anxiety Sensitivity Index; **SUDS** = Subjective Units of Distress Scale (0–100, 0 totally relaxed and 100 the highest distress/anxiety/fear/discomfort ever felt); **CEMS** = Childhood Emotional Manifestation Scale; **mYPAS** = Modified Yale Preoperative Anxiety Scale (23.3–100: the higher the score the higher the anxiety); **STAIC** = State-Trait Anxiety Inventory for Children (20–80: the higher the score the higher the anxiety); **VSA** = Venham Situational Anxiety score; **APPT-WGRS** = Adolescent Pediatric Pain Tool–Word Graphic Rating Scale; **CFS** = McMurtry Children’s Fear Scale; Likert S. =Likert-type scale, with three possible responses (not anxious/anxious, somewhat anxious/anxious or very anxious/anxious); **FLACC** = Face, Legs, Activity, Cry, and Consolability scale; **CHEOPS** = the Children’s Hospital of Eastern Ontario Pain Scale. * Questionnaire or scale that assesses the caregiver or the health professional. ^†^: Did not show whether it was self-administered or hetero-administered. Source: author’s own elaboration.

**Table 7:** GRADE scale.

*Article*	*Evidence*
Gershon, J. et al., 2004 [12]	Low
Gold, J.I. et al., 2006 [15]	Moderate
Chan, E. et al., 2019 [19]	Moderate
Schlechter, A.K. et al., 2021 [20]	Low
Özalp, G. et al., 2020 [21]	Low
Clerc, P.G.B. et al., 2021 [23]	Moderate
Eijlers, R. et al., 2019 [24]	Low
Jung, M.J. et al., 2021 [25]	Moderate
Liu, K.Y. et al., 2021 [26]	Moderate
Jeffs, D. et al., 2014 [27]	Low
Chang, Z.Y. et al., 2022 [30]	Moderate
Goldman, R.D. et al., 2021 [31]	Low

## Data Availability

No new data were created or analyzed in this study. Data sharing is not applicable to this study.

## Supplementary Materials

The following supporting information can be downloaded at: https://www.mdpi.com/article/10.3390/children11080975/s1, S1: PRISMA 2020 Checklist. S2: Rob2.
