# Supplementary material for: Immersive Virtual Reality for Pain and Anxiety Management Associated with Medical Procedures in Children and Adolescents: A Systematic Review
Source: Children (Basel). 2024 Aug 13;11(8):975. doi: 10.3390/children11080975 (PMC11352374; doi:10.3390/children11080975)
Supplement: Supplementary file 1 [file children-11-00975-s001.zip › children-3110166 S2 - Rob2.pdf]

Rob2

	Risk of bias domain					
Article	*D1*	*D2*	*D3*	*D4*	*D5*	***Overall***
Chan, E. et al., 2019 (19)	+	+	+	-	+	-
	+	+	+	-	+	-
Schlechter, A.K. et al., 2021(20)	+	+	?	-	+	-
Özalp, G. et al, 2020 (21)	+	+	?	-	+	-
Gold, J.I. et al., 2006 (15)	+	+	+	-	+	-
Clerc, P.G.B. et al., 2021 (23)	+	+	+	-	+	-
Eijlers, R. et al., 2019 (24)	+	+	?	-	+	-
Jung, M.J. et al., 2021 (25)	+	+	+	-	+	-
Liu, K.Y. et al., 2020 (26)	+	+	+	-	+	-
Jeffs, D. et al., 2014 (27)	+	+	?	-	+	-
Gershon, J. et al., 2004 (12)	+	+	?	-	+	-
Chang, Z.Y. et al., 2022 (30)	+	+	+	-	+	-
Goldman, R.D. et al., 2021 (31)	+	+	?	-	+	-

Domains:
D1: Bias arising from the randomization process.
D2: Bias due to deviations from intended intervention
D3: Bias due to missing outcome data.
D4: Bias in measurement of the outcome
D5: Bias in selection of the reported result.

Judgement:
+ Low
? Some concerns
- High risk
